# Supplementary material for: The Effect of Drenching (Very) Low Birth Weight Piglets with a Dense, Concentrated Milk Replacer at Farms with Differing Farrowing Management
Source: Animals (Basel). 2022 Dec 23;13(1):63. doi: 10.3390/ani13010063 (PMC9817849; doi:10.3390/ani13010063)
Supplement: Supplementary file 1 [file animals-13-00063-s001.zip › animals-2029484-supplementary.pdf]

Supplementary materials

Supplement S1. Cortisol and chromogranin A
Material & Methods

At farm B, saliva was sampled from 30 LBW piglets at the age of 47 days to determine whether sham drenching would induce an acute stress response, and consequently, have an impact on any potential effect of the DMR supplementation. Attempts to collect sufficient volumes of saliva at an earlier age (day 1, day 9, day 27, and day 31 at farm A) were unsuccessful. Saliva samples were analysed to determine the concentration of two known acute stress markers: cortisol and chromogranin A [1]. The LBW piglets were divided into three groups: sham-drenched piglets, piglets that were picked up during 20 sec, and piglets that were snared during 30 sec (positive control) [2]. Saliva samples were collected by picking up the piglets (the animals were not conditioned and would not chew on the pads voluntarily, thus, had to be picked up) and gently inserting a synthetic cylindrical collection pad (MicroSAL, Oasis Diagnostics [3]) into the mouth, before and after the applied treatment. To ensure that the acute stress-induced cortisol release had occurred, saliva samples were collected 30 minutes after the treatment [4]. All samples were kept on ice before being stored at -80 °C until further analysis. The samples were analysed using commercially available ELISA kits for cortisol (IBL-International, RE52611) and Chromogranin A (CgA, MyBioSource, MBS288843) [1].

Statistical analysis

To evaluate the effect of handling piglets during drenching on the cortisol and CgA response, a linear mixed model was used. Treatment, sex, and their interaction were added as fixed factors, and the time between the first and second sampling (immediately before and 30 minutes after the treatment, respectively) was added as a random factor. The model was simplified using the stepwise backward method ($p \leq 0.05$). A *post hoc* analysis was performed using Tukey's correction.

Results

There was no significant interaction between treatment and sex. A treatment effect was observed on the cortisol response ($p = 0.001$). Snaring the LBW piglets resulted in a higher cortisol response, compared to sham-drenching or picking up the animals (Figure S1). No treatment effect was observed for the CgA response ($p = 0.829$) (Figure S1). Sex did not affect the cortisol ($p = 0.765$) or CgA ($p = 0.166$) response.

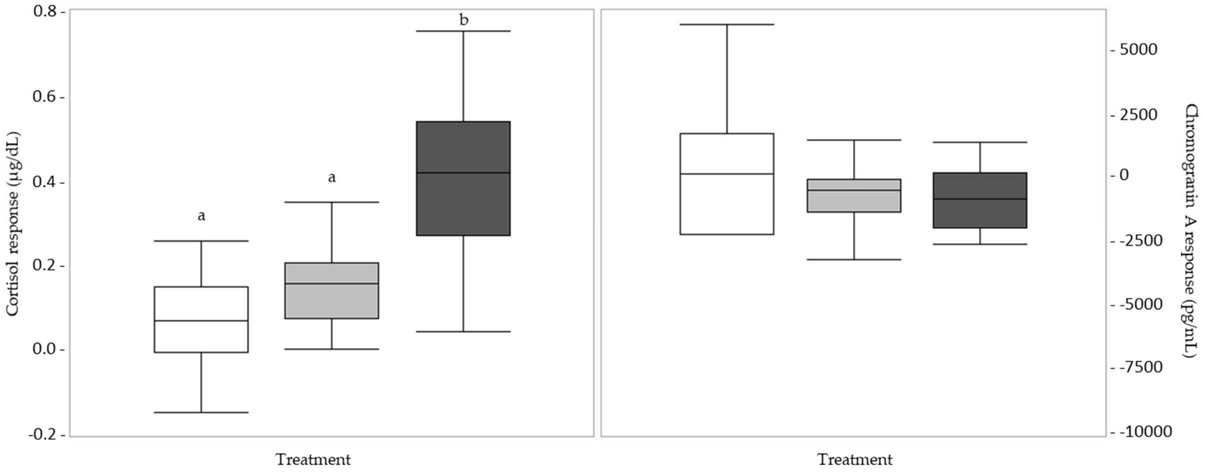

Figure S1. Cortisol response and Chromogranin A (CgA) response in low birth weight piglets after picking up (white box; $n = 10$), sham drenching (light grey box, $n = 10$) or snaring (dark grey box; $n = 10$). Significant differences between treatments are indicated by a different letter.

Table S1. Comparison of body weight, average daily growth (ADG), factorial growth, metabolic weight, factorial metabolic rate and colostrum intake of low birth weight (LBW) and very low birth weight (VLBW) piglets at days 1, 2, 3, 9, and 2 days post-weaning (PW*) at the farm with low (farm A) and high perinatal management (farm B) (median ± SD).

		**FARM A**						**FARM B**		
		VLBW			LBW			LBW		
		Female	Male	VLBW (all)	Female	Male	LBW (all)	Female	Male	LBW (all)
**BODY WEIGHT (KG)**	Day 1	0.67 ± 0.10	0.67 ± 0 10	0.67 ± 0.10	0.87 ± 0.08	0.85 ± 0.08	0.86 ± 0.08	0.88 ± 0.07	0.87 ± 0.06	0.87 ± 0.06
	Day 2	0.69 ± 0.15	0.71 ± 0.14	0.70 ± 0.14	0.93 ± 0.15	0.89 ± 0.19	0.91 ± 0.17	0.89 ± 0.11	0.88 ± 0.09	0.89 ± 0.10
	Day 3	0.80 ± 0.16	0.78 ± 0.20	0.80 ± 0.18	1.03 ± 0.18	0.97 ± 0.20	1.01 ± 0.19	1.02 ± 0.13	0.97 ± 0.12	1.00 ± 0.13
	Day 9	1.66 ± 0.36	1.24 ± 0.38	1.63 ± 0.36	1.99 ± 0.39	1.71 ± 0.45	1.81 ± 0.42	1.88 ± 0.31	1.87 ± 0.28	1.87 ± 0.29
	2 days PW	3.73 ± 0.78	3.07 ± 0.78	3.47 ± 0.77	4.14 ± 0. 97	4.00 ± 0.94	4.02 ± 0.95	4.85 ± 1.00	4.98 ± 1.01	4.90 ± 1.01
**ADG (KG)**	Day 2	0.09 ± 0.18	0.10 ± 0.08	0.10 ± 0.15	0.08 ± 0.10	0.07 ± 0.15	0.08 ± 0.12	0.04 ± 0.09	0.03 ± 0.08	0.04 ± 0.09
	Day 3	0.06 ± 0.09	0.04 ± 0.12	0.10 ± 0.06	0.10 ± 0.10	0.08 ± 0.08	0.10 ± 0.07	0.07 ± 0.05	0.07 ± 0.08	0.07 ± 0.07
	Day 9	0.12 ± 0.05	0.08 ± 0.05	0.11 ± 0.05	0.13 ± 0.05	0.11 ± 0.05	0.12 ± 0.05	0.13 ± 0.04	0.13 ± 0.04	0.13 ± 0.04
	2 days PW	0.13 ± 0.03	0.10 ± 0.03	0.12 ± 0.03	0.15 ± 0.04	0.14 ± 0.04	0.14 ± 0.04	0.15 ± 0.04	0.16 ± 0.04	0.16 ± 0.04
**FACTORIAL GROWTH**	Day 2	1.01 ± 0.27	1.02 ± 0.12	1.02 ± 0.22	1.07 ± 0.14	1.05 ± 0.21	1.06 ± 0.18	1.03 ± 0.11	1.02 ± 0.10	1.02 ± 0.11
	Day 3	1.18 ± 0.33	1.06 ± 0.29	1.14 ± 0.31	1.22 ± 0.18	1.17 ± 0.26	1.20 ± 0.22	1.16 ± 0.13	1.13 ± 0.14	1.16 ± 0.14
	Day 9	2.31 ± 0.66	1.83 ± 0.55	2.25 ± 0.63	2.14 ± 0.59	1.99 ± 0.54	2.12 ± 0.56	2.16 ± 0.34	2.16 ± 0.33	2.16 ± 0.34
	2 days PW	5.47 ± 1.43	4.22 ± 1.09	4.91 ± 1.31	4.95 ± 1.38	4.92 ± 1.44	4.94 ± 1.39	5.29 ± 1.21	5.72 ± 1.19	5.68 ± 1.21
**METABOLIC WEIGHT ($KG^{0.75}$)**	Day 1	0.74 ± 0.09	0.74 ± 0.08	0.74 ± 0.08	0.92 ± 0.05	0.90 ± 0.05	0.89 ± 0.10	0.91 ± 0.05	0.90 ± 0.05	0.90 ± 0.05
	Day 2	0.76 ± 0.13	0.77 ± 0.12	0.77 ± 0.12	0.97 ± 0.09	0.91 ± 0.07	0.93 ± 0.13	0.92 ± 0.08	0.91 ± 0.07	0.92 ± 0.07
	Day 3	0.85 ± 0.12	0.83 ± 0.15	0.85 ± 0.13	1.03 ± 0.09	0.98 ± 0.09	1.01 ± 0.15	1.01 ± 0.10	0.98 ± 0.09	1.00 ± 0.09
	Day 9	1.46 ± 0.24	1.23 ± 0.25	1.44 ± 0.25	1.64 ± 0.39	1.59 ± 0.26	1.56 ± 0.27	1.61 ± 0.20	1.59 ± 0.26	1.59 ± 0.23
	2 days PW	2.68 ± 0.44	2.32 ± 0.44	2.54 ± 0.43	2.90 ± 0.76	3.33 ± 0.51	2.84 ± 0.52	3.27 ± 0.52	3.33 ± 0.51	3.29 ± 0.52
**FACTORIAL METABOLIC WEIGHT**	Day 2	1.01 ± 0.19	1.01 ± 0.09	1.01 ±0.16	1.06 ± 0.07	1.03 ± 0.14	1.05 ± 0.13	1.02 ± 0.08	1.02 ± 0.08	1.02 ± 0.08
	Day 3	1.13 ± 0.22	1.05 ± 0.20	1.11 ± 0.21	1.16 ± 0.08	1.10 ± 0.17	1.15 ± 0.16	1.12 ± 0.09	1.10 ± 0.10	1.11 ± 0.10
	Day 9	1.87 ± 0.40	1.57 ± 0.34	1.84 ± 0.38	1.77 ± 0.41	1.65 ± 0.29	1.76 ± 0.38	1.78 ± 0.21	1.78 ± 0.30	1.78 ± 0.26
	2 days PW	3.58 ± 0.72	2.95 ± 0.56	3.30 ± 0.66	3.27 ± 0.82	3.20 ± 0.84	3.31 ± 0.82	3.49 ± 0.60	3.70 ± 0.58	3.68 ± 0.60
**COLOSTRUM INTAKE (G)**		200.51 ± 156.43	173.78 ± 121.09	199.18 ± 140.30	230.97 ± 76.74	189.83 ± 99.85	223.37 ± 90.57	227.29 ± 97.18	230.58 ± 68.66	227.29 ± 86.77

*2 Days post weaning was on day 24 and day 26 at farm A and B, respectively

Table S2. Comparison of body weight, average daily growth (ADG), factorial growth, metabolic weight, factorial metabolic weight and colostrum intake of low birth weight (LBW) piglets at the farm with low (farm A) and high perinatal care (farm B) for the different treatments (no treatment, sham one dose, sham three doses, dense milk replacer (DMR) one dose and DMR three doses) (median ± SD).

	TREATMENT	FARM A	FARM B	FARM A + B
**BODY WEIGHT**	No treatment	1.01 ± 1.20	0.98 ± 1.60	0.99 ± 1.49
	Sham 1 dose	0.99 ± 1.33	1.02 ± 1.46	1.02 ± 1.42
	Sham 3 doses	1.09 ± 1.22	0.99 ± 1.65	1.01 ± 1.54
	DMR 1 dose	1.04 ± 1.39	1.00 ± 1.42	1.03 ± 1.41
	DMR 3 doses	0.98 ± 1.14	1.02 ± 1.59	0.99 ± 1.47
**ADG (KG)**	No treatment	0.14 ± 0.04	0.16 ± 0.03	0.15 ± 0.04
	Sham 1 dose	0.14 ± 0.04	0.14 ± 0.04	0.14 ± 0.04
	Sham 3 doses	0.14 ± 0.04	0.17 ± 0.03	0.17 ± 0.04
	DMR 1 dose	0.15 ± 0.03	0.15 ± 0.04	0.15 ± 0.04
	DMR 3 doses	0.12 ± 0.04	0.15 ± 0.04	0.15 ± 0.04
**FACTORIAL GROWTH**	No treatment	1.18 ± 1.39	1.13 ± 1.88	1.16 ± 1.74
	Sham 1 dose	1.15 ± 1.55	1.15 ± 1.55	1.15 ± 1.68
	Sham 3 doses	1.16 ± 1.32	1.16 ± 1.32	1.12 ± 1.78
	DMR 1 dose	1.17 ± 1.59	1.17 ± 1.59	1.14 ± 1.62
	DMR 3 doses	1.11 ± 1.27	1.11 ± 1.27	1.12 ± 1.65
**METABOLIC WEIGHT ($KG^{0.75}$)**	No treatment	1.01 ± 0.75	0.98 ± 0.94	0.99 ± 0.88
	Sham 1 dose	0.99 ± 0.82	1.01 ± 0.86	1.01 ± 0.84
	Sham 3 doses	1.07 ± 0.74	0.99 ± 0.95	1.01 ± 0.90
	DMR 1 dose	1.03 ± 0.83	1.00 ± 0.84	1.02 ± 0.84
	DMR 3 doses	0.98 ± 0.71	1.00 ± 0.94	0.98 ± 0.87
**FACTORIAL METABOLIC WEIGHT**	No treatment	1.13 ± 0.83	1.10 ± 1.06	1.11 ± 0.99
	Sham 1 dose	1.11 ± 0.92	1.11 ± 0.98	1.11 ± 0.96
	Sham 3 doses	1.12 ± 0.78	1.08 ± 1.08	1.09 ± 1.00
	DMR 1 dose	1.13 ± 0.92	1.09 ± 0.93	1.10 ± 0.93
	DMR 3 doses	1.08 ± 0.76	1.09 ± 1.02	1.09 ± 0.95
**COLOSTRUM INTAKE (G)**	No treatment	235.87 ± 95.91	279.75 ± 62.69	244.83 91.55
	Sham 1 dose	159.08 ± 108.15	268.89 ± 88.17	204.92 ± 107.19
	Sham 3 doses	130.86 ±36.50	214.57 ±67.71	198.75 ± 65.26
	DMR 1 dose	249.81 ± 38.00	215.18 ± 89.86	231.84 ± 70.93
	DMR 3 doses	240.06 ± 83.10	211.85 ± 1.07.33	240.06 ± 93.45

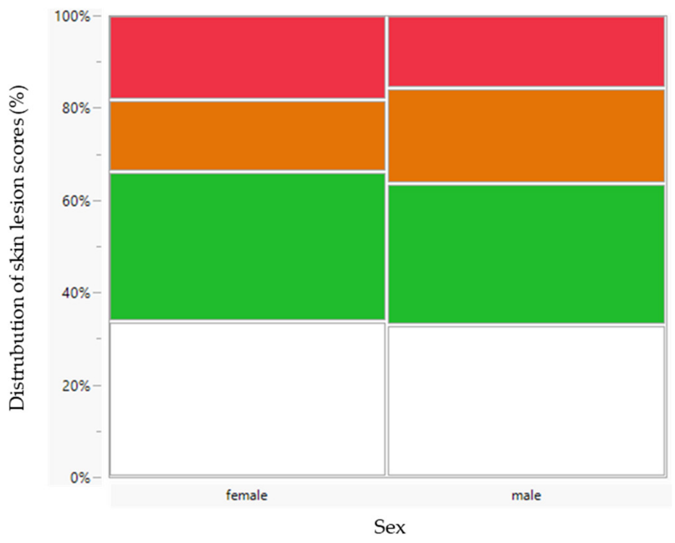

Figure S2. Distribution of skin lesion (SL) scores at farm A (low perinatal management) of the selected low birth weight (LBW) and very low birth weight (VLBW) piglets per sex (female: n = 80; male: n = 80). There was a no significant effect of sex on the probability of having more severe SL. The following scoring system was applied:

0: no lesions (white)

1: < 5 superficial lesions (skin unbroken) (green)

2: 5-10 superficial lesions or < 5 deep lesions (skin broken and evidence of haemorrhage) (orange)

3: > 10 superficial lesions or > 5 deep lesions (red)

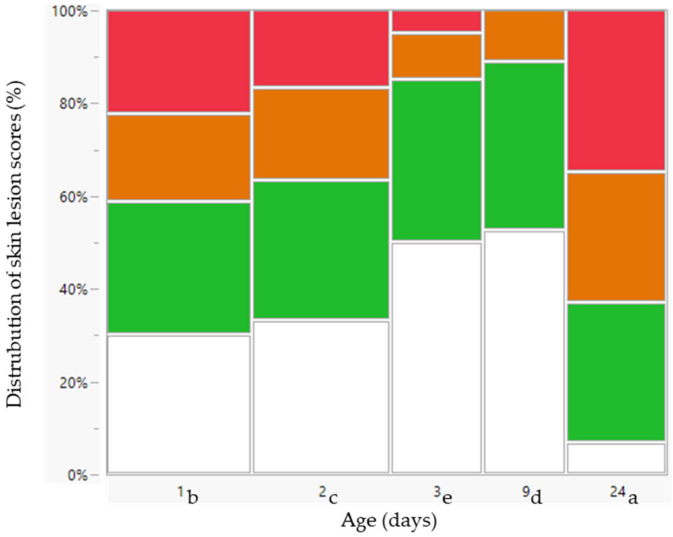

Figure S3. Distribution of skin lesion (SL) scores at farm A (low perinatal management) of the selected low birth weight (LBW, n = 80) and very low birth weight (VLBW, n = 80) piglets per time point. There was a significant age effect on the SL ($p < 0.001$) The probability of having more severe skin lesions is presented by subscripts (from high to low probability, in alphabetical order). The following scoring system was applied:

0: no lesions (white)

1: < 5 superficial lesions (skin unbroken) (green)

2: 5-10 superficial lesions or < 5 deep lesions (skin broken and evidence of haemorrhage) (orange)

3: > 10 superficial lesions or > 5 deep lesions (red)

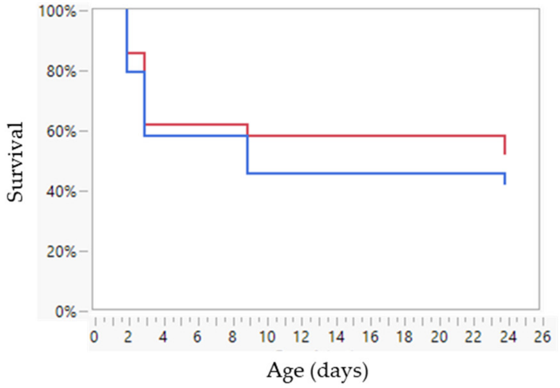

Figure S4. Cumulative mortality of female (red line; n = 80) and male (blue line; n = 80) low birth weight (LBW) and very low birth weight (VLBW) piglets at farm A (low perinatal care). Cox's proportional hazard model showed no sex effect (p = 0.395).

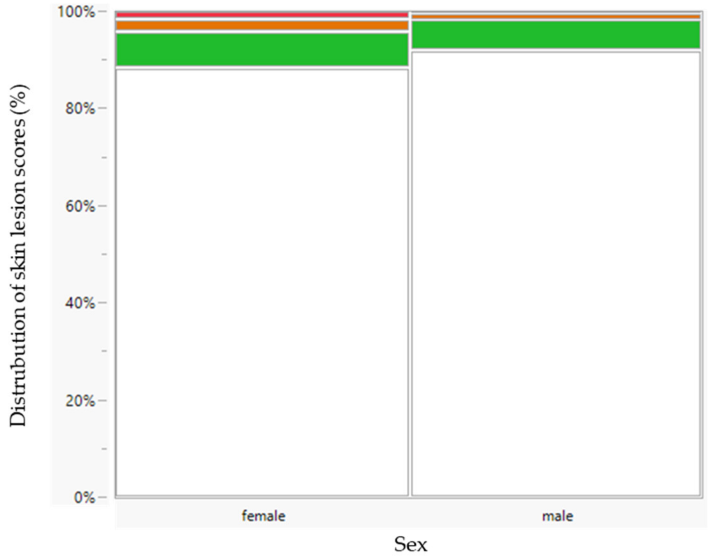

Figure S5. Distribution of skin lesion (SL) scores at farm B (high perinatal management) of the selected low birth weight (LBW) per sex (female: n = 75; male: n = 75). There was a no significant effect of sex on the probability of having more severe SL. The following scoring system was applied:

0: no lesions (white)

1: < 5 superficial lesions (skin unbroken) (green)

2: 5-10 superficial lesions or < 5 deep lesions (skin broken and evidence of haemorrhage) (orange)

3: > 10 superficial lesions or > 5 deep lesions (red)

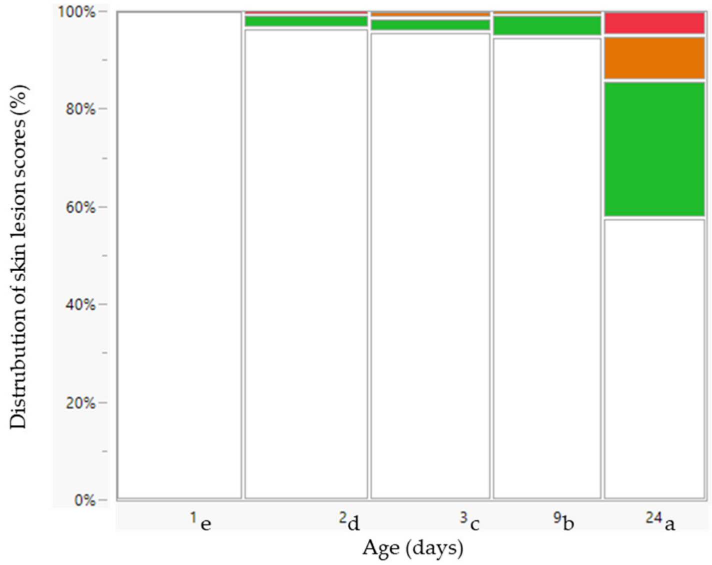

Figure S6. Distribution of skin lesion (SL) scores at farm B (high perinatal management) of the selected low birth weight (LBW, n = 150) piglets per time point. There was a significant age effect on the SL (p < 0.001) The probability of having more severe skin lesions is presented by subscripts (from high to low probability, in alphabetical order). The following scoring system was applied:

0: no lesions (white)

1: < 5 superficial lesions (skin unbroken) (green)

2: 5-10 superficial lesions or < 5 deep lesions (skin broken and evidence of haemorrhage) (orange)

3: > 10 superficial lesions or > 5 deep lesions (red)

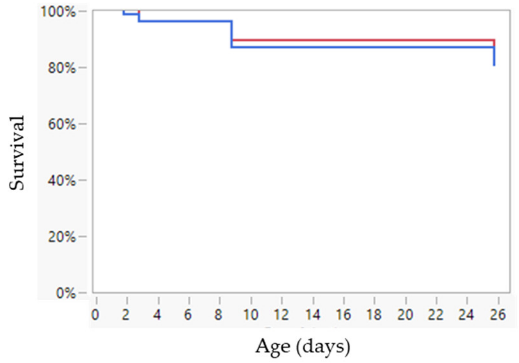

Figure S7. Cumulative mortality of female (red line; n = 75) and male (blue line; n = 75) low birth weight (LBW) piglets at farm B (high perinatal care). Cox's proportional hazard model showed no sex effect (p = 0.886).

References

1. Escribano, D.; Ko, H.L.; Chong, Q.; Llonch, L.; Manteca, X.; Llonch, P. Salivary biomarkers to monitor stress due to aggression after weaning in piglets. *Res Vet Sci* **2019**, *123*, 178-183, doi:10.1016/j.rvsc.2019.01.014.
2. Escribano, D.; Soler, L.; Gutiérrez, A.M.; Martínez-Subiela, S.; Cerón, J.J. Measurement of chromogranin A in porcine saliva: validation of a time-resolved immunofluorometric assay and evaluation of its application as a marker of acute stress. *Animal* **2013**, *7*, 640-647, doi:10.1017/s1751731112002005.
3. Shirtcliff, E.A.; Buck, R.L.; Laughlin, M.J.; Hart, T.; Cole, C.R.; Slowey, P.D. Salivary Cortisol Results Obtainable Within Minutes of Sample Collection Correspond With Traditional Immunoassays. *Clinical Therapeutics* **2015**, *37*, 505-514, doi:https://doi.org/10.1016/j.clinthera.2015.02.014.
4. Bozovic, D.; Racic, M.; Ivkovic, N. Salivary cortisol levels as a biological marker of stress reaction. *Med Arch* **2013**, *67*, 374-377, doi:10.5455/medarh.2013.67.374-377.
